# Hydrous magnesium-rich magma genesis at the top of the lower mantle

**DOI:** 10.1038/s41598-019-43949-2

**Published:** 2019-05-15

**Authors:** Ayano Nakajima, Tatsuya Sakamaki, Takaaki Kawazoe, Akio Suzuki

**Affiliations:** 1 The Division of Earth and Planetary Materials Science, Tohoku University, 6-3, Aoba, Aramaki, Aoba-ku, Sendai 980-8578 Japan; 2 Bayerisches Geoinstitut, University of Bayreuth, 95440 Bayreuth, Germany; 3 Department of Earth and Planetary Systems Science, Hiroshima University, Higashi-Hiroshima, 739-8526 Japan

**Keywords:** Planetary science, Solid Earth sciences

## Abstract

Several igneous activities occur on the surface of the Earth, including island arcs, mid-ocean ridges and hot spots. Based on geophysical observations, melting phenomena in the interior also occur at the asthenosphere’s top and the upper mantle’s bottom. Additionally, a seismological low-velocity anomaly was observed at the top of the lower mantle that may result from mantle melting due to dehydration decomposition of ringwoodite to bridgmanite and ferropericlase with a downward flow. However, the corresponding high-pressure experimental data are too poor to understand the melting phenomena under the lower mantle condition. Herein, we conducted hydrous peridotite melting experiments at pressures from 23.5 to 26 GPa and at temperatures from 1300 to 1600 °C for demonstrating the melt composition and the gravitational stability of magma at the top of the lower mantle. The melt had a SiO_2_-poor and MgO-rich composition, which is completely different than that of dry peridotite melting experiments. Compared with the seismological lower mantle, the experimental melt is gravitationally lighter; thus, a similar melt could be observed as seismological low-velocity zone at the lower mantle’s top. The generated magma plays as a filter of down-welling mantle and can contribute to a formation of a silicate perovskitic lower mantle.

## Introduction

Hydrous ringwoodite with about 1.5 wt.% H_2_O^1^ and phase egg^2–4^ has been discovered in the diamond inclusions of mantle xenoliths. Ringwoodite is a high-pressure polymorph of olivine and a significant mineral in the mantle transition zone. Phase egg is one of several hydrous aluminosilicate minerals which is stable under the conditions found in the mantle transition zone^2,3^. Identifying these hydrous minerals of the mantle transition zone would be natural evidence supporting the hypothesis that the mantle transition zone is a water reservoir.

As wadsleyite and ringwoodite are major constituent minerals of the mantle transition zone, their water capacities have been experimentally investigated. The water solubilities of wadsleyite and ringwoodite have been estimated to be about 1–3 wt.%^5,6^. As a basis of comparison, the water solubilities of major constituent minerals of the mantle, olivine and bridgmanite, are about 0.1 wt.%^6^ and 0.2 wt.%^7^, respectively.

The water-rich mantle transition zone plays an important role as a trigger of mantle melting. Owing to the large difference in water solubilities between olivine and wadsleyite, dehydration melting can occur at the 410 km discontinuity at the beginning of the transition zone^8^. Geophysical observations support the existence of melt at the base of the upper mantle based on low seismic wave velocity anomalies^9,10^ and high electrical conductivity^10^. In addition, the gravitational stability of hydrous magma was experimentally confirmed by Sakamaki *et al*.^11^. However, water-induced mantle melting can occur not only above the mantle transition zone but also below the transition zone. Significantly, a low-velocity anomaly has been detected at the top of the lower mantle^12–14^.

Melting experiments on mantle materials have been widely performed^15–17^. The composition of partial melt becomes magnesium (Mg)-rich and silicon (Si)-poor with increasing pressure^15^. In the case of melting under the lower mantle condition, the liquidus phase is bridgmanite, and the composition of the coexisting melt is richer in iron (Fe) and Calcium (Ca) than that in bridgmanite^16,18–21^. Kawamoto conducted experiments with a water-saturated KLB-1 peridotite melt at 14–24 GPa and 900 °C–1400 °C^22^. A clear difference was observed in crystal-melt partitioning between the dry and hydrous systems. The melt exhibited a Ca-rich and Si-, Mg- and aluminium (Al)-poor composition as compared with the anhydrous system. A problem with this experiment was the valence of iron in the starting materials. Although samples with divalent iron (Fe^2+^) have generally been used in previous studies, trivalent iron (Fe^3+^) is more dominant at the top of the lower mantle^23,24^. This is because the mineral structure of bridgmanite is such that it easily accepts an Fe^3+^ and at least 60–80% of iron cations are Fe^3+^ ^24,25^. Melting experiments of this type should be performed using the more dominant trivalent iron.

Herein, we conducted melting experiments with a hydrous peridotite composition with Fe^3+^ to more accurately reproduce the melting phenomenon that occurs at the boundary between the mantle transition zone and the lower mantle.

## Results

### Chemical compositions of partial melts and obtained crystals

**Figure 1:**
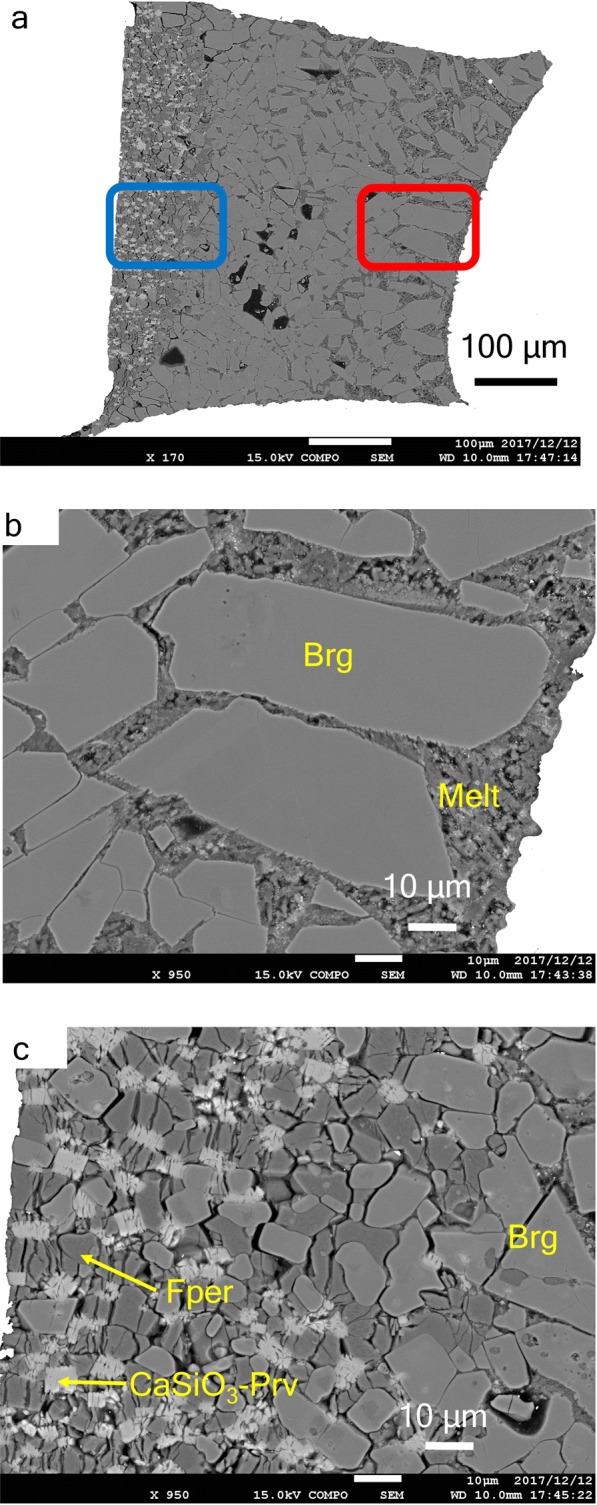
Photographs of the recovered sample at 26 GPa and 1400 °C (duration 120 min). (**a**) Scanning electron microscopy (SEM) image of the entire sample. The right is the center side of heater and high-temperature region. (**b**) Back-scatter detector (BSE) image of the high-temperature side (red area in **a**). (**c**) BSE image of the low-temperature side (blue area in **a**). Brg, Fper and CaSiO_3_-Prv denote bridgmanite, ferropericlace and CaSiO_3_-perovskite, respectively. The grey coloured crystals are bridgmanite, the dark black ones are ferropericlase and the light grey ones are CaSiO_3_-perovskite.

Hydrous peridotite melting experiments were conducted at 1300 °C–1600 °C at 23.5 and 26 GPa. All recovered samples contained bridgmanite, CaSiO_3_-perovskite and ferropericlase with quench microcrystals from the melt. This implies that all experiments were performed between solidus and liquidus (Table 1 and Supplementary Fig. S1). A cross section of representative experimental products (26 GPa and 1400 °C for 120 min) is shown in Fig. 1. The longer the duration of the experiment, the larger the crystals grew. We evaluate the chemical equilibrium of the sample based on experiments with different retention times (60 min and 120 min) at the same experimental condition (P = 26 GPa and T = 1400 °C). Since the compositional difference fell within the standard error range, we regarded melting experiments as equilibrium (Supplementary Tables S1.5 and S1.6). The difference in crystal distribution can be clearly seen in Fig. 1a. Most crystals were bridgmanite. In Fig. 1b (=high-temperature side), microcrystals around larger bridgmanite crystals can be observed. On the lower temperature side, ferropericlase and CaSiO_3_-perovskite have crystallised (Fig. 1c).

**Table 1:** Experimental conditions and crystallization order.

P (GPa)	T (°C)	Duration (min)	Crystals
23.5	1400	50	melt → Bg → CaSiO_3_-Prv, Fper
23.5	1500	60	melt → Bg → CaSiO_3_-Prv, Fper
23.5	1600	15	melt → Bg → CaSiO_3_-Prv, Fper
26.0	1300*	100	melt → Bg → CaSiO_3_-Prv, Fper
26.0	1400	60	melt → Bg → CaSiO_3_-Prv, Fper
26.0	1400	120	melt → Bg → CaSiO_3_-Prv, Fper
26.0	1500	30	melt → Bg → CaSiO_3_-Prv, Fper
26.0	1590	15	melt → Bg → CaSiO_3_-Prv Fper

Bridgmanite, CaSiO_3_-perovskite and ferropericlase are shown by Brg, CaSiO_3_-Prv and Fper, respectively. The temperature value denoted by an asterisk was estimated based on supplying electrical power due to breaking of thermocouple.

**Figure 2:**
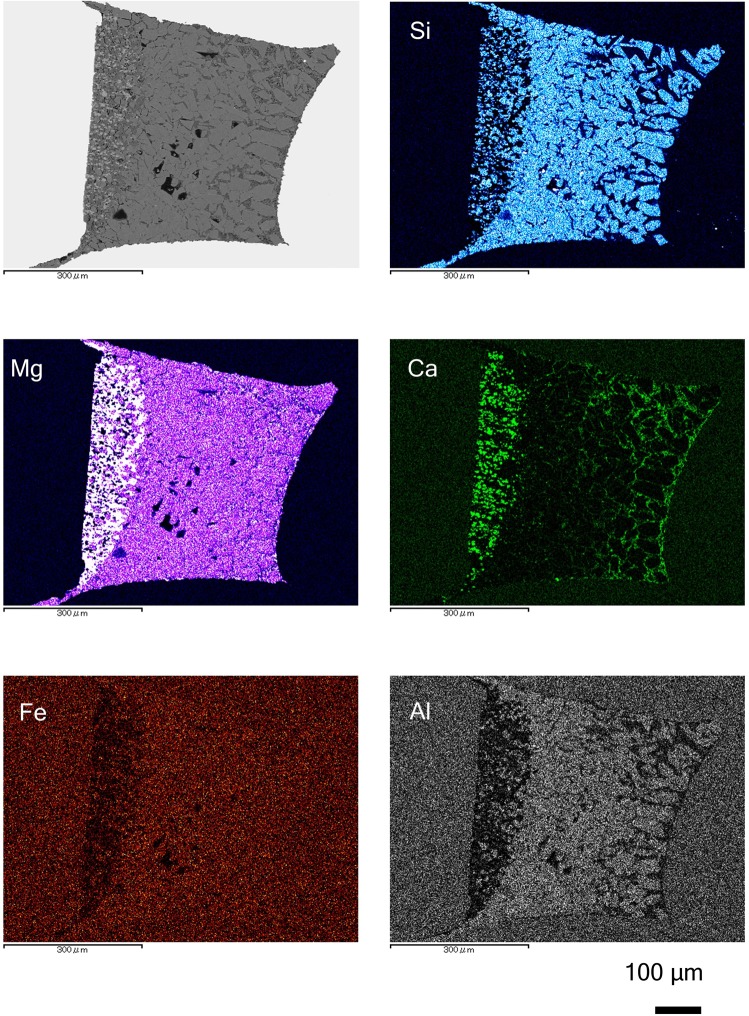
Chemical mapping images of recovered sample at 26 GPa and 1400 °C (120-min duration). An upper left picture is the electron microscope image, and the others are mapping data of silicon (Si), magnesium (Mg), calcium (Ca), iron (Fe) and aluminium (Al). Higher concentration of elements corresponds to the bright areas of the image. Ghost peaks in gold capsule are shown in Fe- and Al-mapping images.

**Figure 3:**
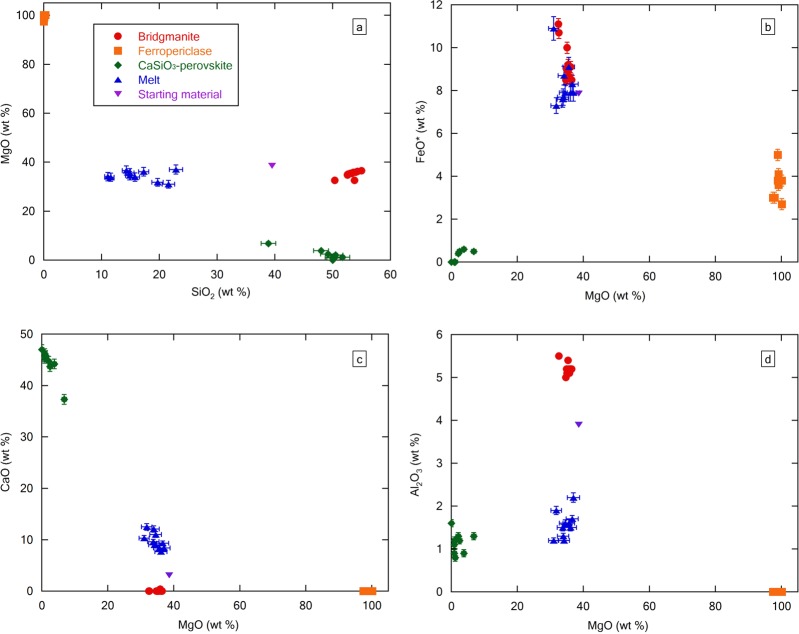
Chemical composition of crystals and melt in the recovered sample. (**a**) Plot of the SiO_2_–MgO ratio of each crystal and melt. (**b**) Plot of the MgO–FeO ratio of each crystal and melt. Iron oxides are calculated as Fe^3+^ except for ferropericlase. (**c**) Plot of the MgO–Al_2_O_3_ ratio of each crystal and melt. (**d**) Plot of the MgO–CaO ratio of each crystal and melt. Error bars are standard errors.

Chemical mapping images of the recovered sample are shown in Fig. 2 and Supplementary Fig. S2. The brightest area in the Si mapping is bridgmanite; Mg is ferropericlase; Ca is CaSiO_3_-perovskite; Fe is bridgmanite and the melt; and Al is bridgmanite. In the Fe-mapping, the dark region is consistent with an existence of CaSiO_3_-perovskite. The ratios of each component are plotted in Fig. 3. The SiO_2_ content of the melt is less than half that of bridgmanite and CaSiO_3_-perovskite (Fig. 3a), and the MgO and FeO contents of bridgmanite and the melt are nearly identical (Fig. 3b). The melt contains a higher CaO concentration than the bridgmanite (Fig. 3c), and the bridgmanite exhibits the highest Al_2_O_3_ content (Fig. 3d) among all samples. The composition of bridgmanite was 53.5 ± 0.8 wt.% SiO_2_, 5.2 ± 0.1 wt.% Al_2_O_3_, 9.0 ± 0.4 wt.% FeO* and 35.5 ± 0.6 wt.% MgO. Total cations were calculated as Fe^3+^ except ferropericlase (Supplementary Table S1). The cation ratio of recovered sample was estimated based on stoichiometry of analyzed chemical composition using energy dispersive X-ray spectrometry (EDS). Based on the estimation, bridgmanite was mostly fitted with Fe^3+^, and ferropericlase was accommodated to Fe^2+^. This result implies that the bridgmanite^26^ contains little Fe^2+^, and ferropericlase comprises mainly Fe^2+^. The averaged composition of the partial melt was 15.8 ± 3.5 wt.% SiO_2_, 1.6 ± 0.3 wt.% Al_2_O_3_, 8.0 ± 0.5 wt.% FeO*, 34.8 ± 1.6 wt.% MgO and 9.8 ± 1.6 wt.% CaO. These values represent the average composition of each measurement of melt shown in Supplementary Table S1 and the uncertainties are calculated from the standard deviation. Comparison with anhydrous experiments, the partial melt had a CaO-rich and SiO_2_- and Al_2_O_3_-poor composition. Notably, the weight content of SiO_2_ was only one half that of MgO. The atomic ratio of Mg/Si for this study is 4.3, whereas that of the anhydrous melt is 1.0^18^; Kawamoto^22^ calculated the atomic ratio of Mg/Si of the hydrous melt as only 1.9 in a previous hydrous study (Table 2). Such high-MgO and low-SiO_2_-content melt can influence density and compressibility under deep mantle conditions.

**Table 2:** Comparison of chemical compositions of bridgmanite and melt with other previous experiments.

	Hydrous				Anhydrous			
	Bridgmanite (this study)	Melt (this study)	Bridgmanite (Kawamoto^22^)	Melt (Kawamoto^22^)	Bridgmanite (Ito & Takahashi^18^)	Melt (Ito & Takahashi^18^)	Bridgmanite (Trønnes & Frost^19^)	Melt (Trønnes & Frost^19^)
Pressure (GPa)	26		24		25		24.5	
Temperature (°C)	1400		1400		~2500		~2400	
SiO_2_ (wt.%)	53.6 (0.5)	11.6 (3.5)	54.54	17.87	54.6 (0.8)	40.9 (0.3)	54.4	46
Al_2_O_3_ (wt.%)	5.1 (0.3)	1.5 (0.4)	4.65	1.04	3.2 (0.2)	3.3 (0.1)	3.73	4.03
FeO (wt.%)	9.1 (0.7)	7.6 (0.6)	5.24	4.91	3.6 (0.6)	10.2 (0.2)	3.98	8.43
MgO (wt.%)	35.8 (0.6)	33.8 (1.8)	35.92	23.28	36.5 (1.0)	31.9 (0.6)	36.1	35.5
CaO (wt.%)	—	9.6 (2.7)	0.13	12.59	0.42 (0.04)	8.9 (0.3)	0.86	4.42
Mg#	0.887	0.898	0.92	0.89	0.964	0.847	0.942	0.883
Mg/Si	0.996	4.344	0.98	1.94	0.888	1.007	0.989	1.150
Fe_melt_/Fe_Brg_	0.89		0.94		2.83		2.12	

The composition of iron oxides is calculated as ferrous iron except for this study.

### Comparing the chemical composition of crystal and the partial melt

When comparing the composition of bridgmanite with that of melts, no difference was observed in MgO content (Fig. 3; Supplementary Table S1). This trend is similar to the results of previous experiments performed with anhydrous systems (Table 2). The important point is that less difference in iron content between melt and bridgmanite (Fe_melt_/Fe_Brg_ = 0.9). Compared to anhydrous system (Fe_melt_/Fe_Brg_ = 2–3)^18,19^, preferred concentration of Fe into melt is not observed. In the case of melting experiment of Fe^2+^-bearing sample^22^, the Fe content ratio of bridgmanite to melt is also about 0.9 (see Table 2). The Fe-Mg exchange partition ${K}_{D}^{(Fe/Mg)}$ between bridgmanite and melt of anhydrous peridotite is about 0.4^18,19^, while that of this study (hydrous system) is 1.1.

### Density of the melt

To discuss the gravitational stability of the melt in the lower mantle, the melt density was calculated using Eq. (1) proposed by Wakabayashi & Funamori^27^.

1 $$P=\frac{3}{2}{K}_{0,{T}_{0}}({(\frac{{V}_{0,{T}_{0}}}{{V}_{p,T}})}^{\frac{7}{3}}-{(\frac{{V}_{0,{T}_{0}}}{{V}_{P,T}})}^{\frac{5}{3}})+{\alpha }_{0,{T}_{0}}{K}_{0,{T}_{0}}(T-{T}_{0})$$

In Eq. (1), *P*, *K*, *V*, *α* and *T* represent pressure, bulk modulus, volume, the thermal expansion coefficient and temperature, respectively. Because water concentrates in the melt of run products, considering the effect of H_2_O on the density of silicate melts is necessary. The partial molar volume of H_2_O in magma at high pressure and temperature was calculated using the Eq. (2):

2 $$P=3{K}_{T}[1-{(\frac{{{\nabla }}_{{H}_{2}O}}{{{\nabla }}_{{H}_{2}O,0}})}^{\frac{1}{3}}]{(\frac{{{\nabla }}_{{H}_{2}O}}{{{\nabla }}_{{H}_{2}O,0}})}^{\frac{2}{3}}\exp \{\frac{3}{2}(K^{\prime} -1)[1-{(\frac{{{\nabla }}_{{H}_{2}O}}{{{\nabla }}_{{H}_{2}O,0}})}^{\frac{1}{3}}]\}\,$$

where ${{\nabla }}_{{H}_{2}O}$ is the high-pressure partial molar volume of H_2_O, ${{\nabla }}_{{H}_{2}O,0}$ is the zero-pressure partial molar volume as described by Bouhifd *et al*.^28^ and *K*_*T*_ is the isothermal bulk modulus as described by Sakamaki^29^.

**Figure 4:**
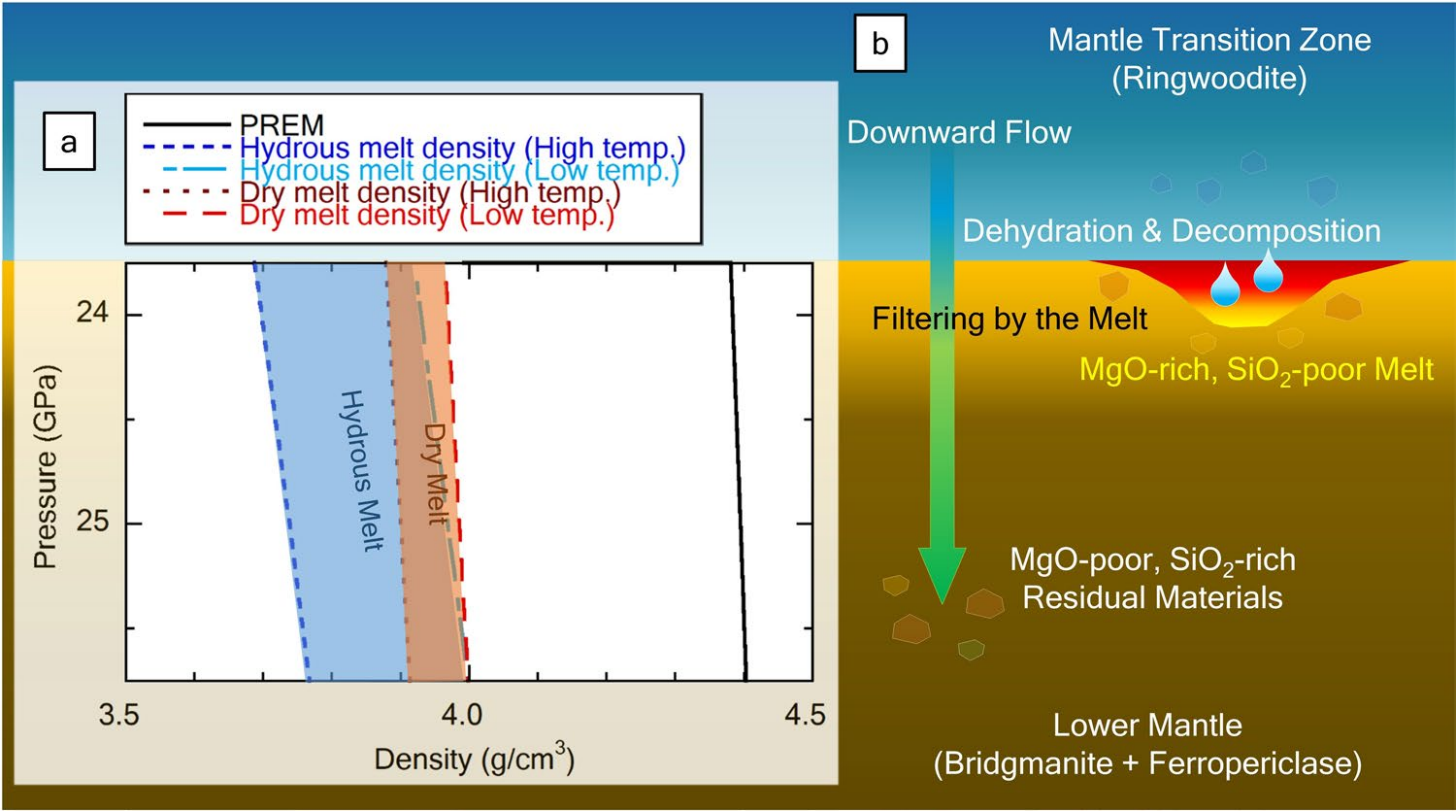
(**a**) Compression curves of the calculated melt under the condition of top of lower mantle. The preliminary reference earth model (PREM) was plotted based on Dziewonski & Anderson^30^. The densities of dry and hydrous melts are calculated based on the equations given by Wakabayashi & Funamori^27^ and Sakamaki^29^ along the mantle geotherm. Low-temperature densities are represented based on Brown and Shankland^32^, while high-temperature data using Stacey^33^. (**b**) Schematic illustration of the dehydration melting at the top of lower mantle. Downward mantle convection causes dehydration decomposition and generation of hydrous melt, which is MgO-rich and SiO_2_-poor composition. Residual rocks are low Mg/Si ratio and sinking deeper with gravitationally separating from the melt. The peridotitic lower mantle can be formed by the residual materials.

Considering the values in Supplementary Table 1, the total value of the melt is low (64.0–78.3 wt.%). Owing to the feature of EDS, measurement of light elements, such as H, is impossible; moreover, if H is concentrated in the sample, the average of the total value (wt.%) is estimated to be low. It is not conclusive what leads to the low total value because other influences such as vacancy must also be considered, but coexisting solid phases are anhydrous minerals; hence, we estimated that the remaining weight ratio of the melt was concentrated water (see Supplementary Fig. S4 for detail). We calculated the chemical compositions of hydrous magma based on the average composition of obtained results and the water content of 29.9 ± 4.1 wt.%. The compression curve of the obtained melt is calculated by the extrapolation of the Eqs (1) and (2). Figure 4a shows two calculated patterns of hydrous melt (29.9 ± 4.1 wt.% H_2_O) and dry melt (0 wt.% H_2_O). Comparing with the preliminary reference earth model (PREM)^30^, both melt densities of this study become lighter than the lower mantle. This means that the melt in this study would be gravitationally stable around the top of the lower mantle regardless of the melt’s water content.

## Discussions

The mantle transition zone can act as a water reservoir/source, and a hydrous condition can be created at the top of the lower mantle. The solidus of peridotite rocks under anhydrous conditions is much higher than that under the mantle geotherm^16,18,19,21^. However, the presence of water lowers the melting point of the rock, and as a result, the rock begins to produce a partial melt. This MgO-rich and SiO_2_-poor melt becomes lighter owing to the higher concentrations of water and lower concentrations of iron. Thus, the generated melt at the top of the lower mantle can be separated from the surrounding mantle (Fig. 4b) and be observed as a low velocity anomaly^12–14^. This melt is caused by a downward flow such as a slab^13,14^ and may react with the mantle transition zone. Even if this melt is absorbed by the mantle transition zone, the melt can be continuously generated at the top of lower mantle as long as the down-welling mantle continues.

The residual mantle rocks separate from the melt due to the large density difference. The downward mantle rock is poor in melt component, that is, the lower mantle composition below the melt can be MgO-poor and SiO_2_-rich, which is consistent with silicate perovskitic composition. The silicate perovskitic lower mantle has been proposed based on the comparison of experimentally determined sound velocity of lower mantle minerals with seismological observation^31^. The melt at the top of lower mantle plays as a filter and causes a chemical contrast between upper and lower mantles. Our results can provide a possible mechanism for formation of the silicate perovskitic lower mantle.

## Methods

### Starting material

Reagent powders (SiO_2_, Al_2_O_3_, Fe_2_O_3_, MgO and CaCO_3_) were mixed and heated for decarbonation of CaCO_3_ at 1000 °C for 16 h. Then, brucite (Mg(OH)_2_) was added to the decarbonated powder. The water content was 6.98 wt.% (Table 3), and #Mg = Mg/(Mg + Fe) = 0.907.

**Table 3:** Chemical composition of the starting material based on McDonough *et al*.^34^.

	wt.%	mol%
Si	18.5	11.7
Al	2.07	1.37
Fe	5.50	1.76
Mg	23.3	17.1
Ca	2.22	0.991
H	0.781	13.8
O	47.7	53.2
total	100	100.0
SiO_2_	39.5	37.4
Al_2_O_3_	3.90	2.18
Fe_2_O_3_	7.87	2.80
MgO	38.6	32.5
CaO	3.11	3.16
H_2_O	**6.98**	22.0
total	100.0	100.0

The ratio of ferrous iron to ferric one (Fe^2+^/Fe^3+^) is 0 in the starting material.

### High pressure and temperature experiment

High pressure and temperature experiments were conducted using a Kawai-type multi-anvil press (Hymag 1,000-tonne press) at Bayerisches Geoinstitut (BGI) in Germany. We used a 32-mm second-stage anvil (ha7%Co/hawedia) and a 7/3 cell assembly. The sample was enclosed in a gold capsule. Supplementary Fig. S4 shows a detailed design of the cell assembly.

Pressure and temperature conditions were 23.5 and 26 GPa and 1300, 1400, 1500 and 1600 (or 1590) °C, respectively (Table 1 and Supplementary Fig. S1). In the experiment where the duration was doubled (60 min became 120 min) at 26 GPa and 1400 °C, crystal growth was observed; however, no significant difference was observed in chemical composition. After maintaining the temperature for 15 to 120 min, the samples were recovered via quenching.

### Analysis of the recovered samples

Recovered samples were polished using sand papers and abrasives ranging from #80 to #3000, and then finished with 3-μm and 1-μm diamond paste. Field emission scanning electron microscope (FE-SEM; JSM-7001F/JEOL) and EDS (SN: 60154/Oxford Instruments) instruments were used to analyse the samples. The acceleration voltage was 15.0 kV, and the emission current was 77–80 μA.

We estimated the Fe^3+^/Fe^2+^ ratio of minerals in the recovered sample based on stoichiometry of analyzed chemical composition using EDS. Based on the estimation, bridgmanite is Fe^3+^-rich and ferropericlase is Fe^2+^-rich. However, we could not determine the Fe^3+^/Fe^2+^ ratio of the melt. The EDS measurements of melt composition have been performed under the same analysis conditions. The melt composition shown in the Supplementary Table S1 takes the average of ten times measurements.

### Calculations of melt density

The reference temperature *T*_0_ was 2500 K, and the temperature of the required density *T* was set to 1873 K. Consequently, calculations yielded that ${K}_{0,{T}_{0}}$, ${V}_{0,{T}_{0}}$ and ${\alpha }_{0,{T}_{0}}$ are 64.4 GPa, 17.0 cm^3^/mol and 15.4 × 10^−5^/K, respectively. In the case of dry peridotitc melt, these parameters were calculated to be 54 GPa, 18.0 cm^3^/mol and 11.5 × 10^−5^/K, respectively^27^.

In Eq. (1), it is assumed that this is in an anhydrous system, and the Fe valence is perfectly Fe^2+^ (=FeO). However, we used Fe_2_O_3_ in this study.

## Supplementary information

Supplementary Information

## Data Availability

The data used to obtain the conclusions in this study are provided in the main article and the Supplementary Information.
