# Supplementary material for: Hydrous magnesium-rich magma genesis at the top of the lower mantle
Source: Sci Rep. 2019 May 15;9:7420. doi: 10.1038/s41598-019-43949-2 (PMC6520349; doi:10.1038/s41598-019-43949-2)
Supplement: Supplementary file 1 — Supplementary Information [file 41598_2019_43949_MOESM1_ESM.pdf]

Hydrous magnesium-rich magma genesis at the top of the lower mantle

Ayano Nakajima[1*], Tatsuya Sakamaki[1], Takaaki Kawazoe[2] & Akio Suzuki[1]

[1] *The Division of Earth and Planetary Materials Science, Tohoku University, 6-3, Aoba, Aramaki, Aoba-ku, Sendai 980-8578, Japan.*

[2] *Bayerisches Geoinstitut, University of Bayreuth, 95440 Bayreuth, Germany.*

(*ayano.nakajima.r1@dc.tohoku.ac.jp).

Supplementary Information

Supplementary Figure: 17 figures

Supplementary Tables: 8 tables

Supplementary References: 5 references

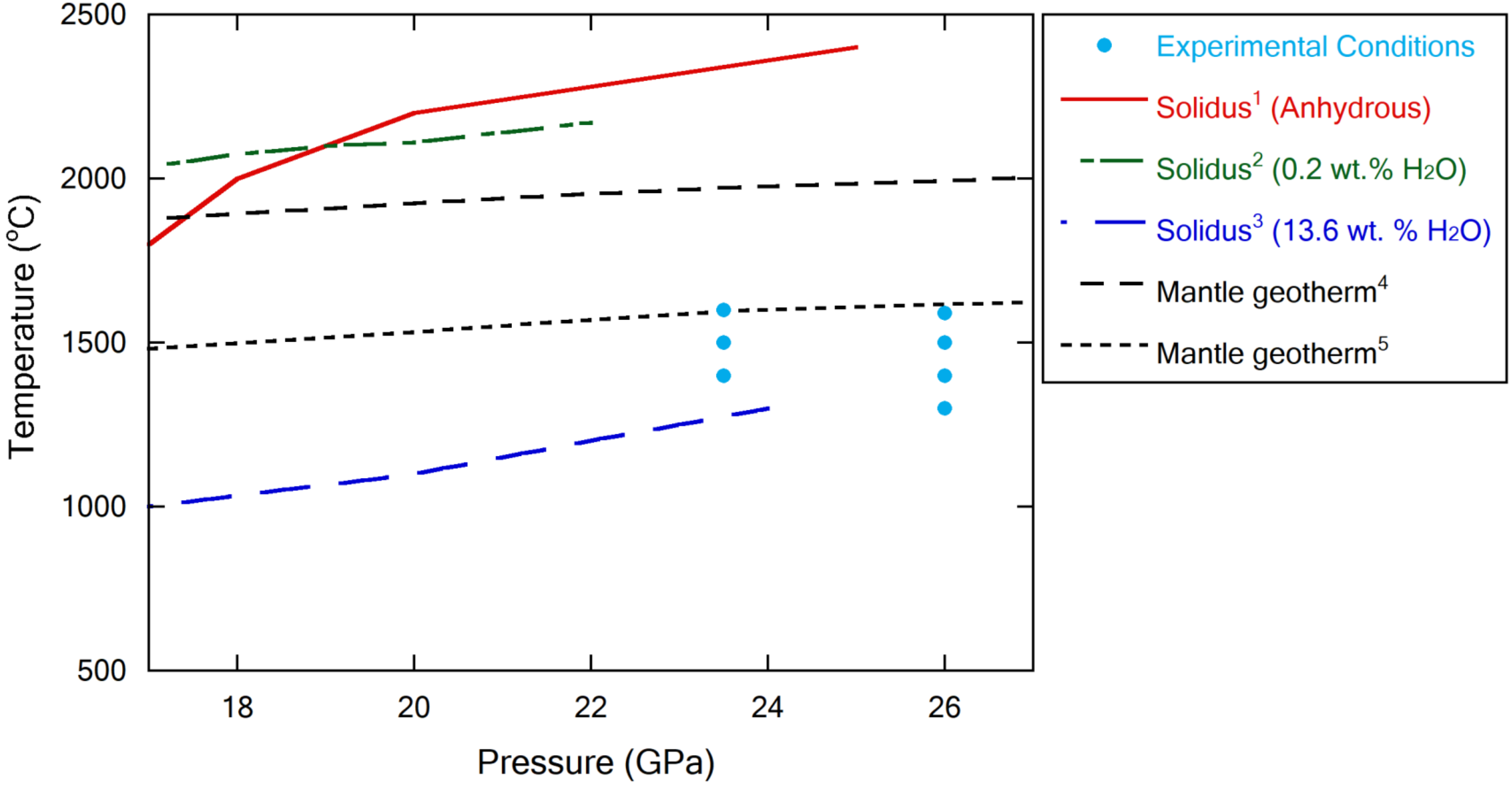

Supplementary Figure S1. Pressure and temperature conditions of this study (light blue points).
Red line is a solidus temperature of anhydrous peridotite conducted by Ito & Takahashi[1]. Green and blue lines are solidus temperatures of 0.2 wt. % H_2O and 13.6 wt.% H_2O peridotite given by Iwamori[2] and Kawamoto[3], respectively. Broken and dashed lines are mantle geotherm by Stacey[4] and Brown & Shankland[5], respectively.

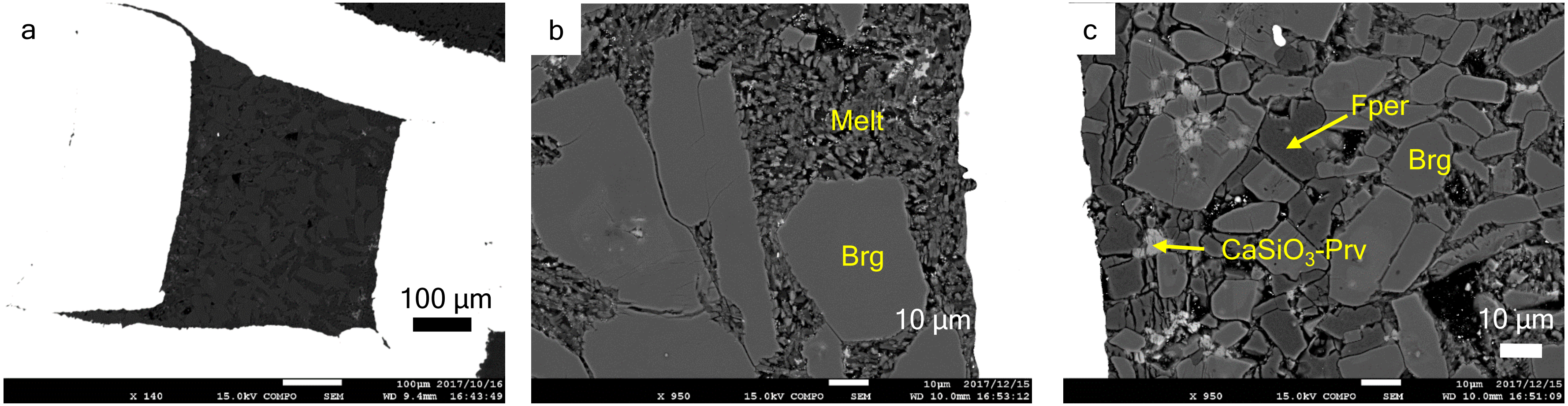

Supplementary Figure S2.1. Photographs of recovered sample at 23.5 GPa and 1400 °C (duration time: 50 minutes). (a) Whole SEM BSE image. The right side is thermocouple (high temperature) side. (b) BSE image of low temperature side. (c) BSE image of high temperature side. Brg, Fper and $CaSiO_3$-Prv indicate bridgmanite, ferropericlase and $CaSiO_3$-perovskite, respectively.

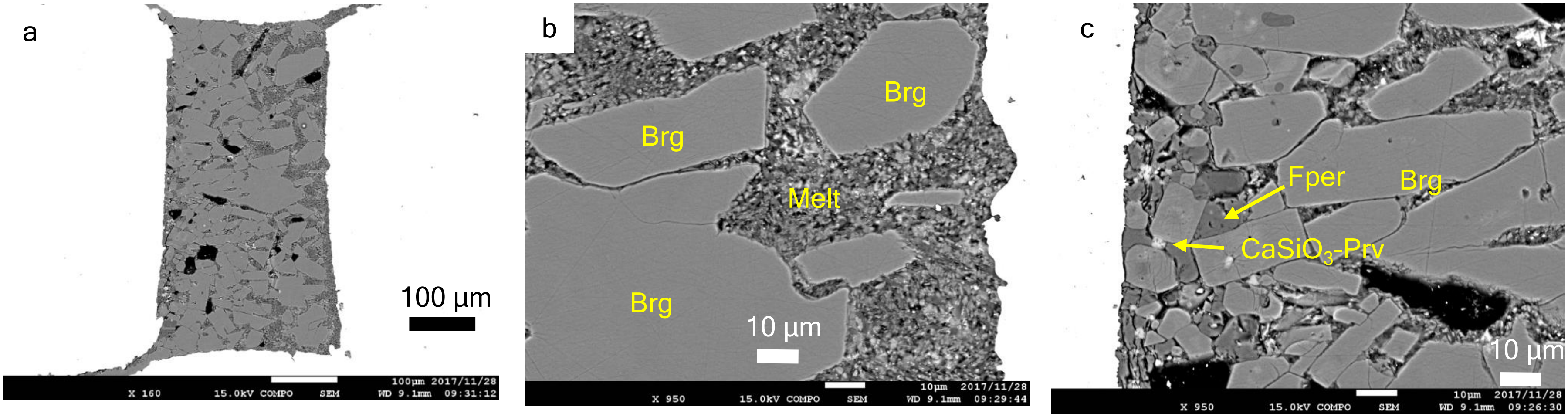

Supplementary Figure S2.2. Photographs of recovered sample at 23.5 GPa and 1500 °C (duration time: 60 minutes). (a) Whole SEM BSE image. The right side is thermocouple (high temperature) side. (b) BSE image of low temperature side. (c) BSE image of high temperature side.

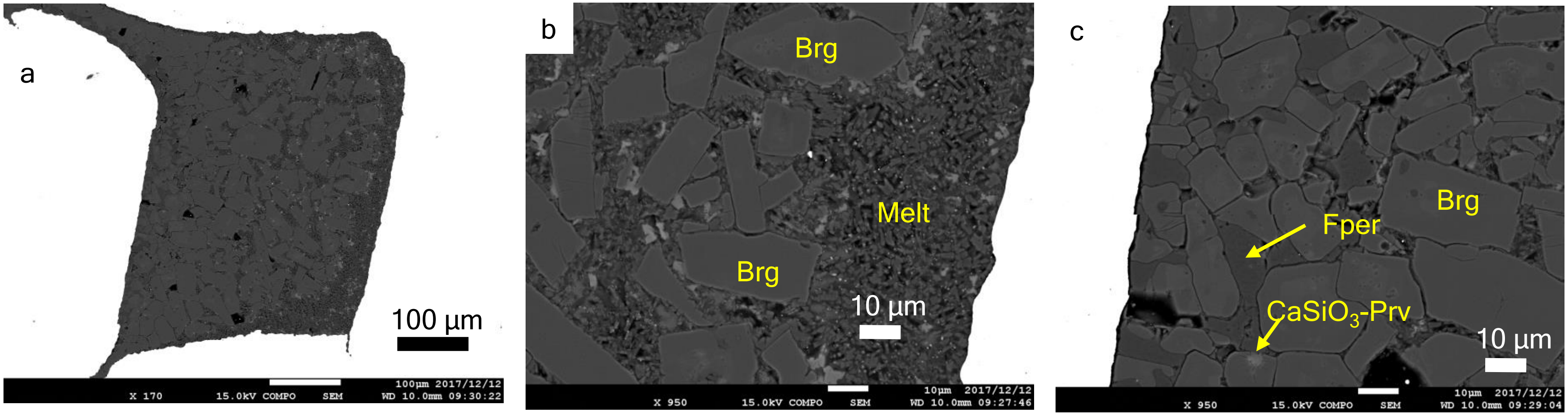

Supplementary Figure S2.3. Photographs of recovered sample at 23.5 GPa and 1600 °C (duration time: 15 minutes). (a) Whole SEM BSE image. The right side is thermocouple (high temperature) side. (b) BSE image of low temperature side. (c) BSE image of high temperature side.

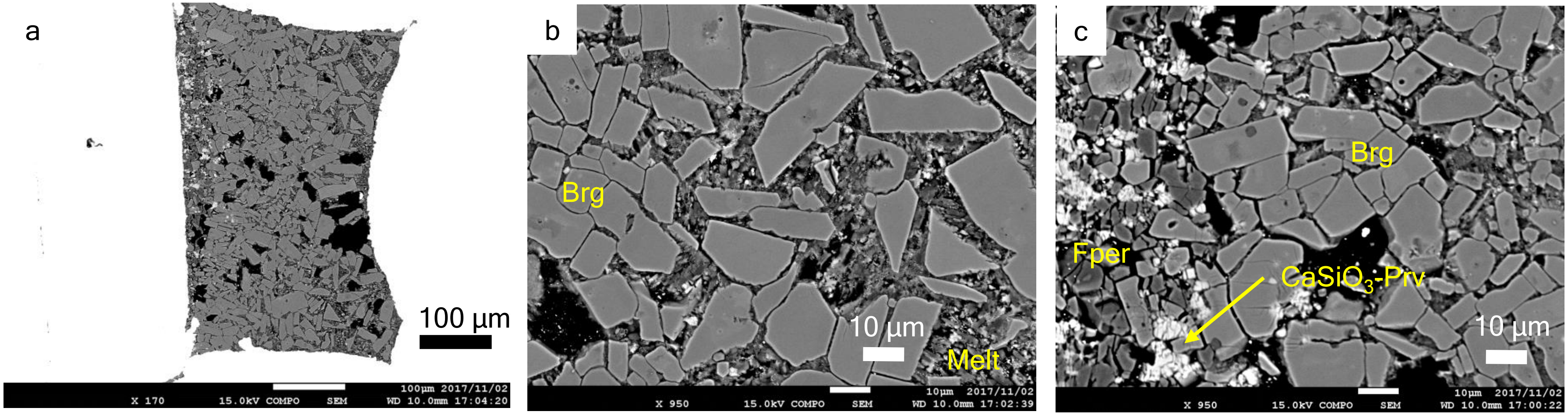

Supplementary Figure S2.4. Photographs of recovered sample at 26 GPa and 1300 °C* (duration time: 100 minutes).
(a) Whole SEM BSE image. The right side is thermocouple (high temperature) side. (b) BSE image of low temperature side.
(c) BSE image of high temperature side.

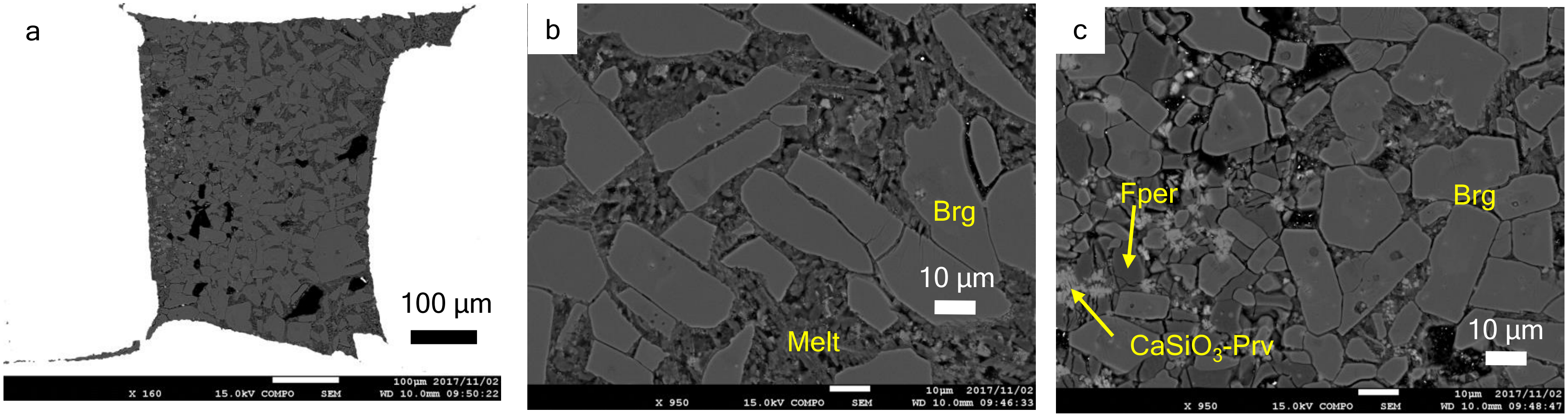

Supplementary Figure S2.5. Photographs of recovered sample at 26 GPa and 1400 °C (duration time: 60 minutes). (a) Whole SEM BSE image. The right side is thermocouple (high temperature) side. (b) BSE image of low temperature side. (c) BSE image of high temperature side.

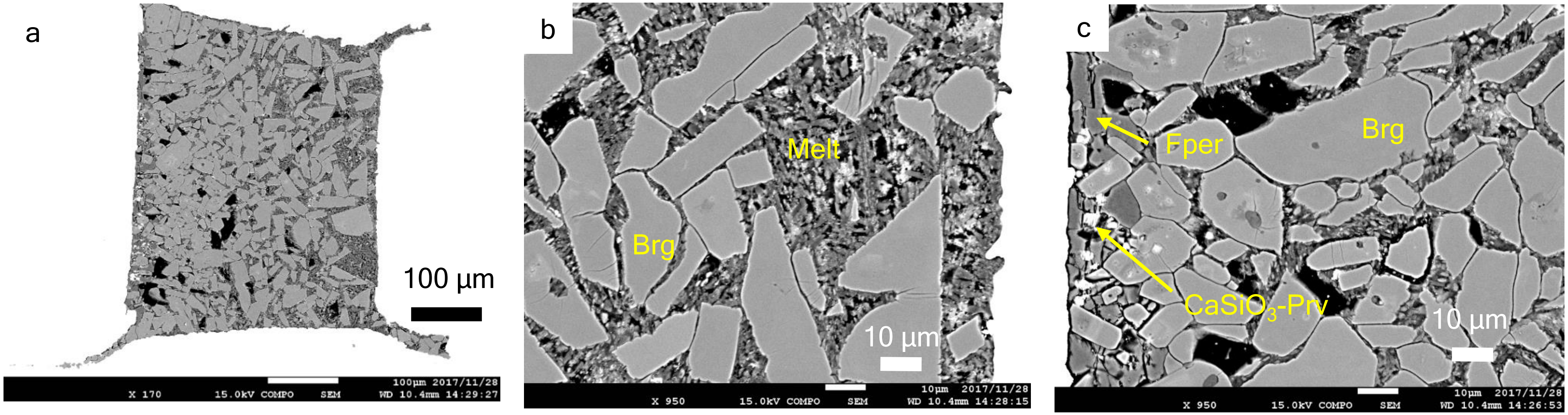

Supplementary Figure S2.6. Photographs of recovered sample at 26 GPa and 1500 °C (duration time: 30 minutes).
(a) Whole SEM BSE image. The right side is thermocouple (high temperature) side. (b) BSE image of low temperature side.
(c) BSE image of high temperature side.

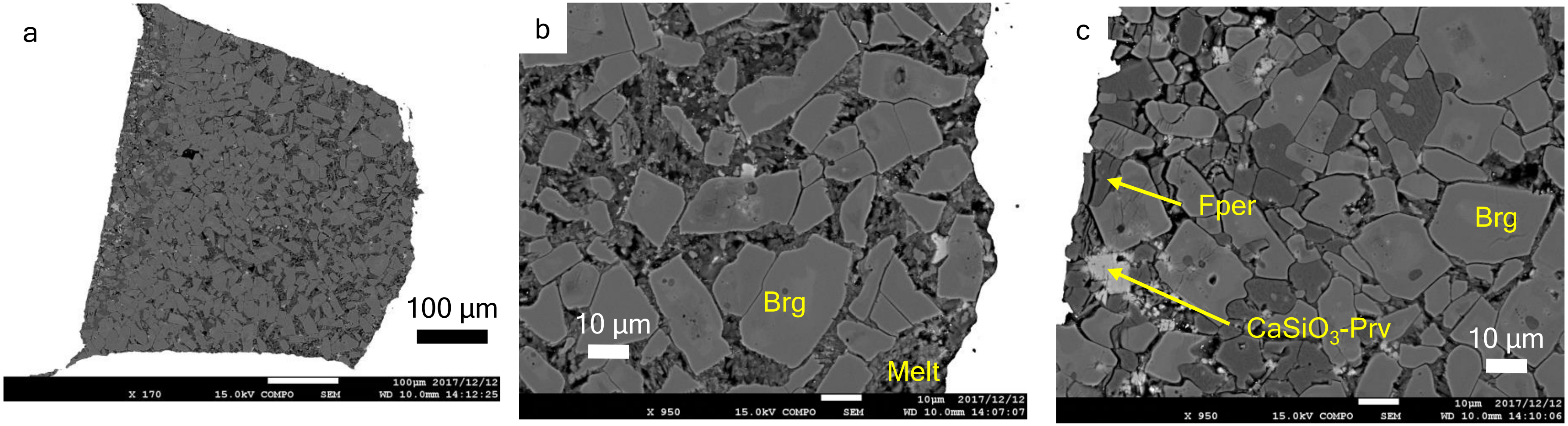

Supplementary Figure S2.7. Photographs of recovered sample at 26 GPa and 1590 °C (duration time: 15 minutes).
(a) Whole SEM BSE image. The right side is thermocouple (high temperature) side. (b) BSE image of low temperature side.
(c) BSE image of high temperature side.

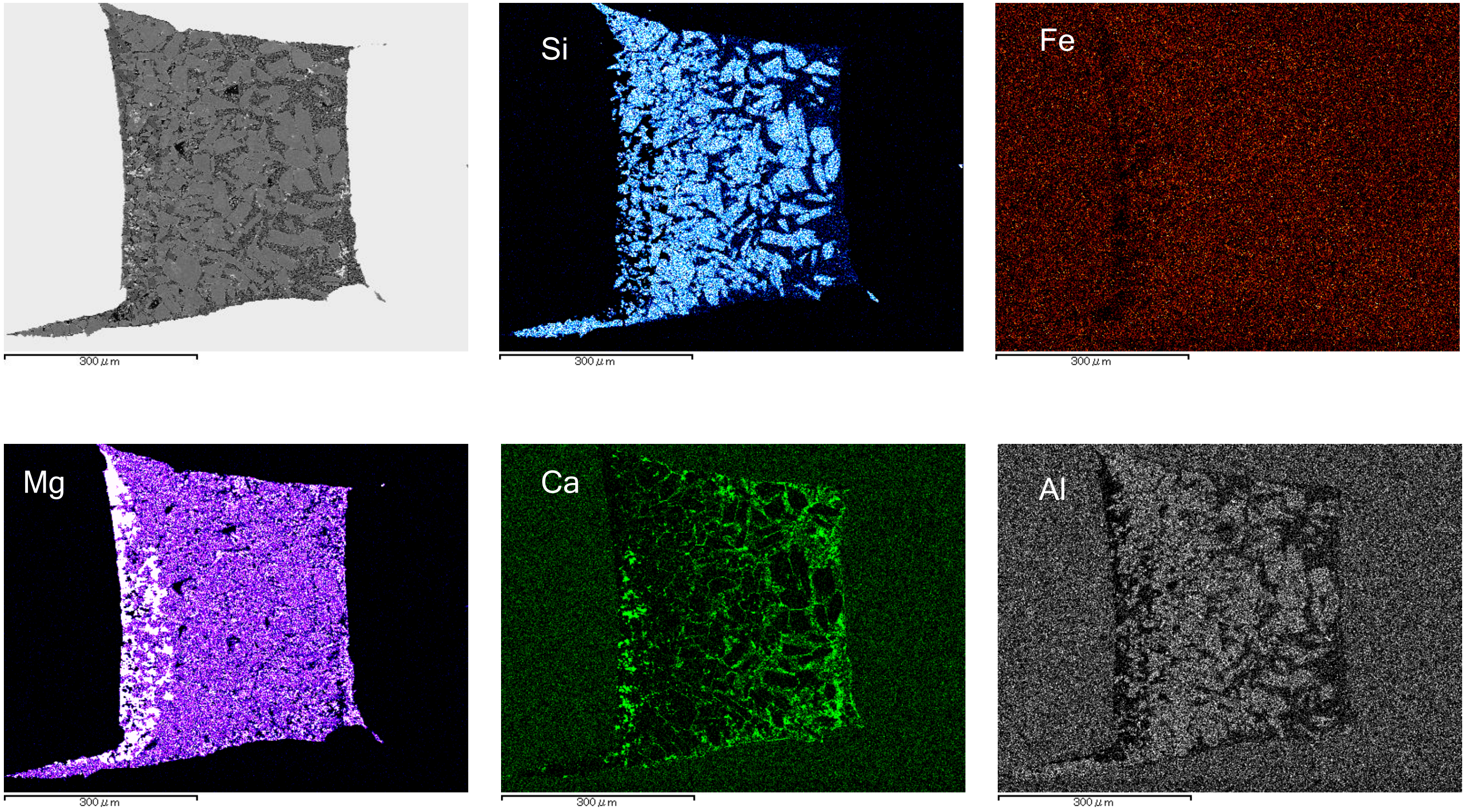

Supplementary Figure S3.1. Elemental mapping images and electron microscope image (upper left) of recovered sample at 23.5 GPa and 1400 °C (duration time: 50 minutes).

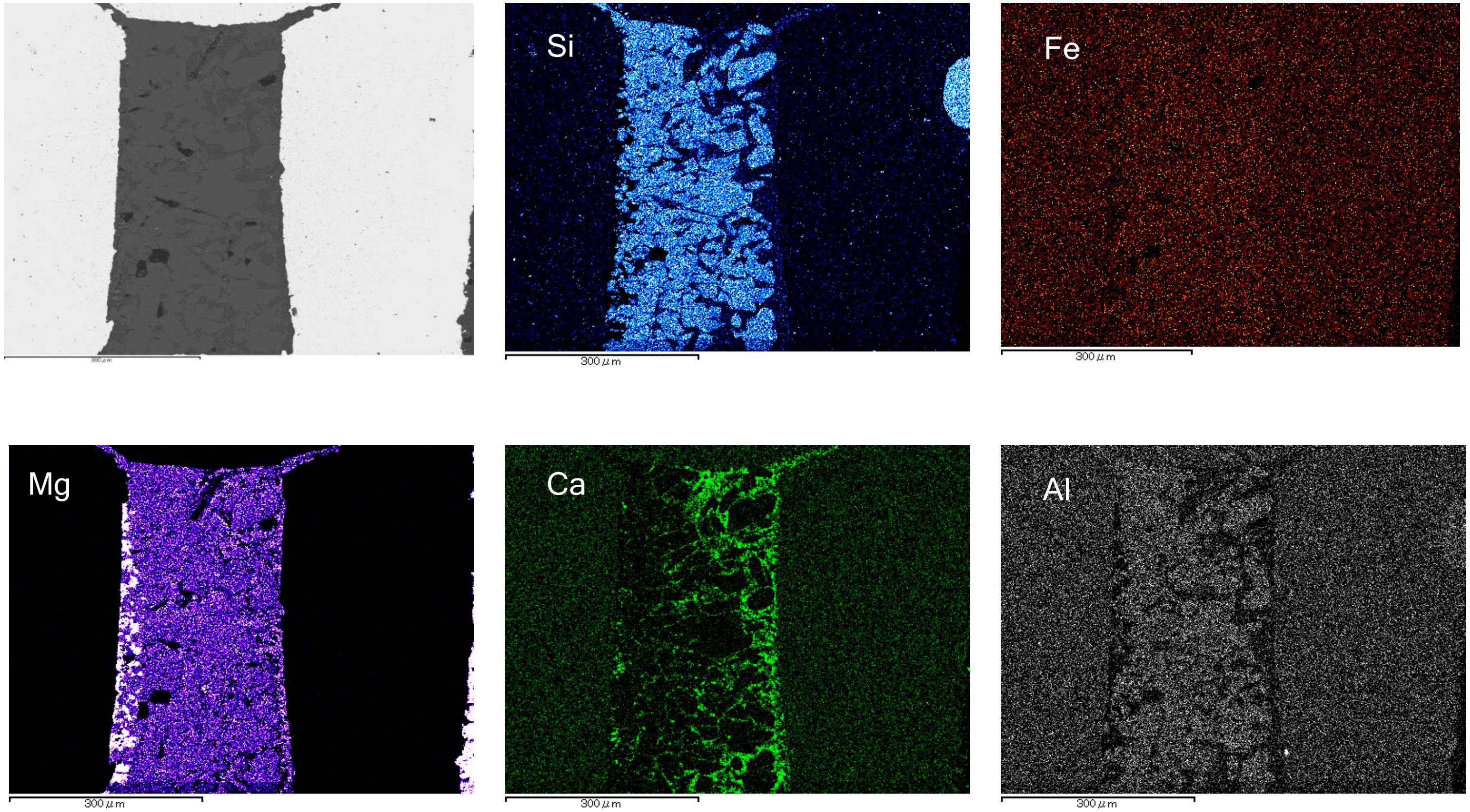

Supplementary Figure S3.2. Elemental mapping images and electron microscope image (upper left) of recovered sample at 23.5 GPa and 1500 °C (duration time: 60 minutes).

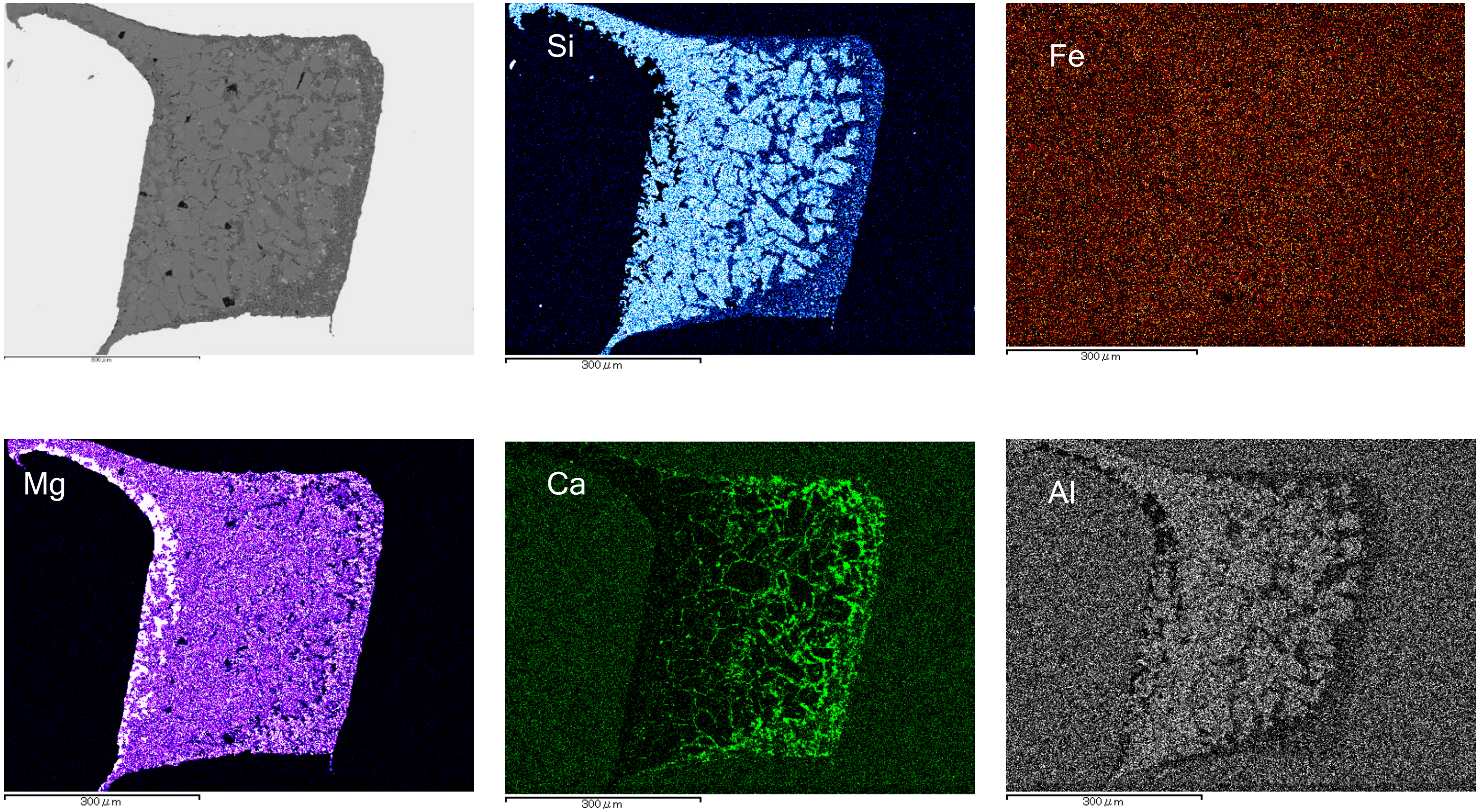

Supplementary Figure S3.3. Elemental mapping images and electron microscope image (upper left) of recovered sample at 23.5 GPa and 1600 °C (duration time: 15 minutes).

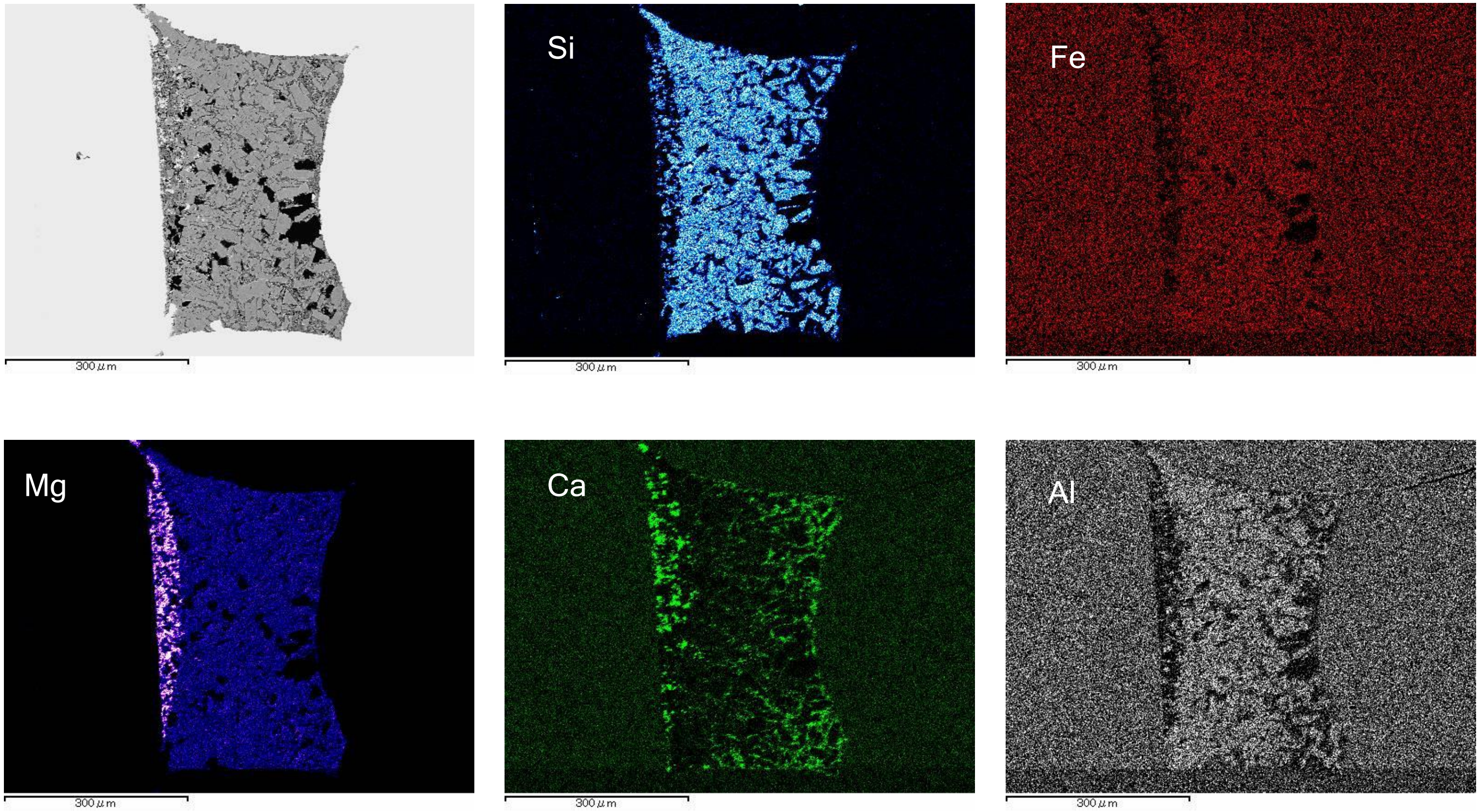

Supplementary Figure S3.4. Elemental mapping images and electron microscope image (upper left) of recovered sample at 26 GPa and 1300 °C* (duration time: 100 minutes).

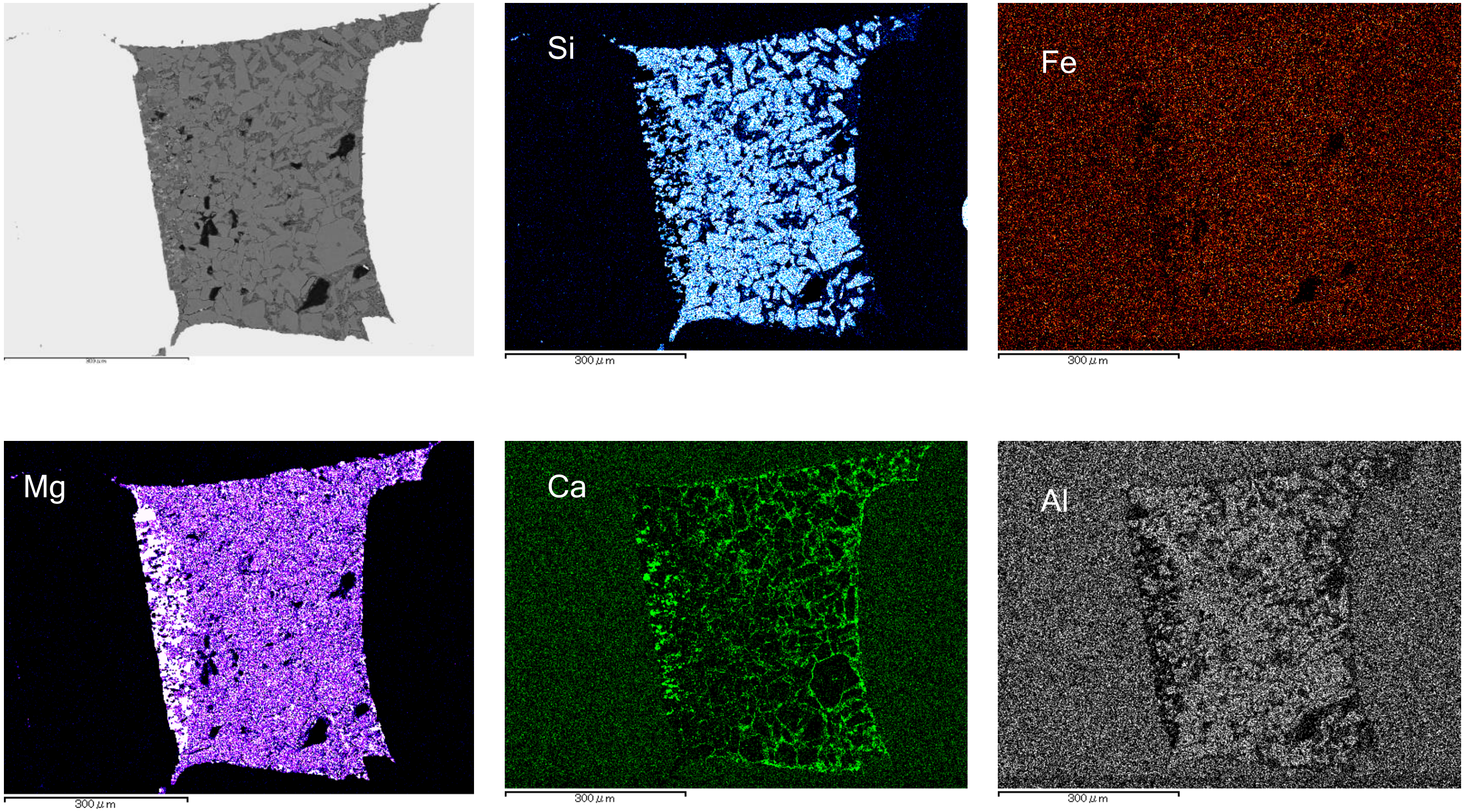

Supplementary Figure S3.5. Elemental mapping images and electron microscope image (upper left) of recovered sample at 26 GPa and 1400 °C (duration time: 60 minutes).

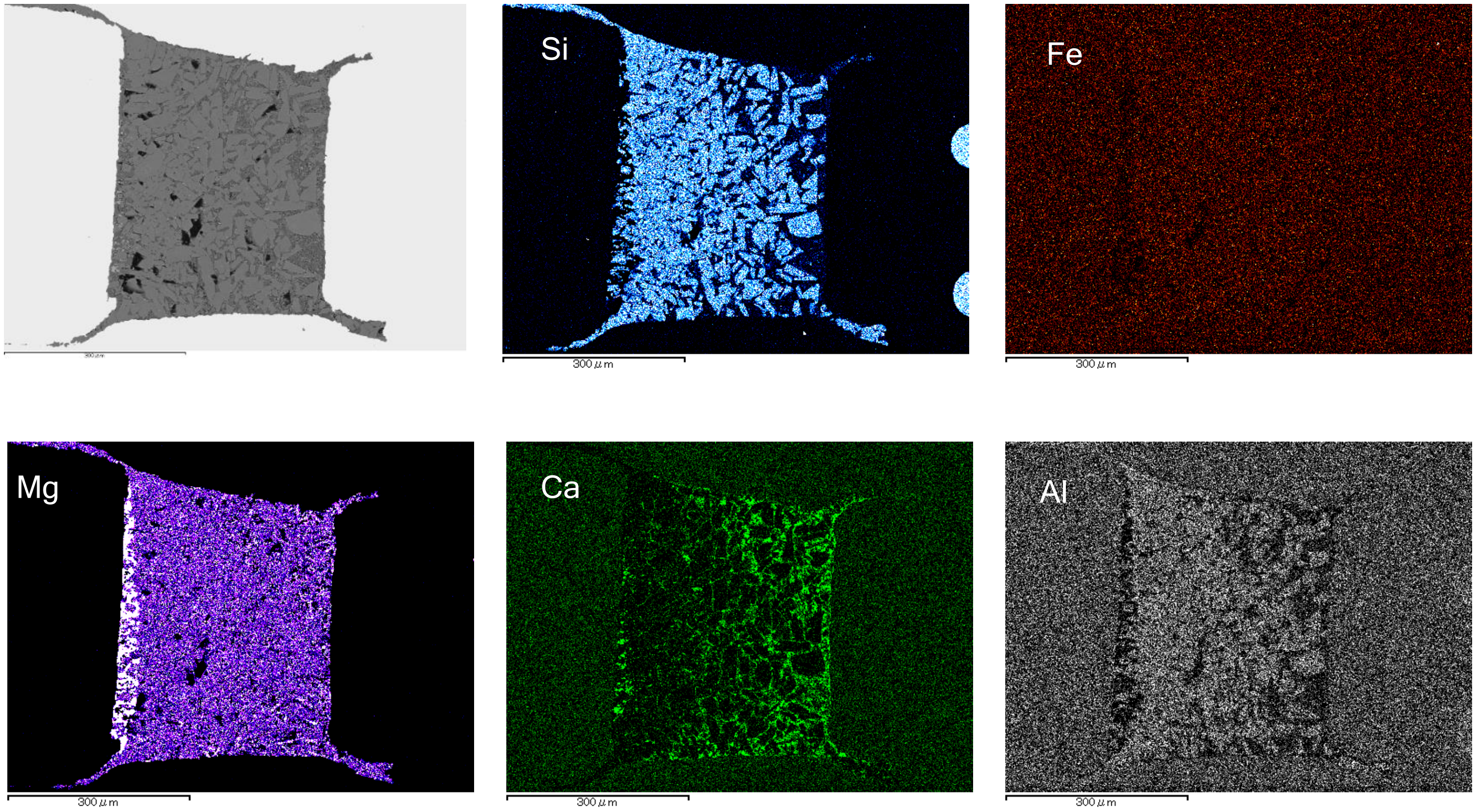

Supplementary Figure S3.6. Elemental mapping images and electron microscope image (upper left) of recovered sample at 26 GPa and 1500 °C (duration time: 30 minutes).

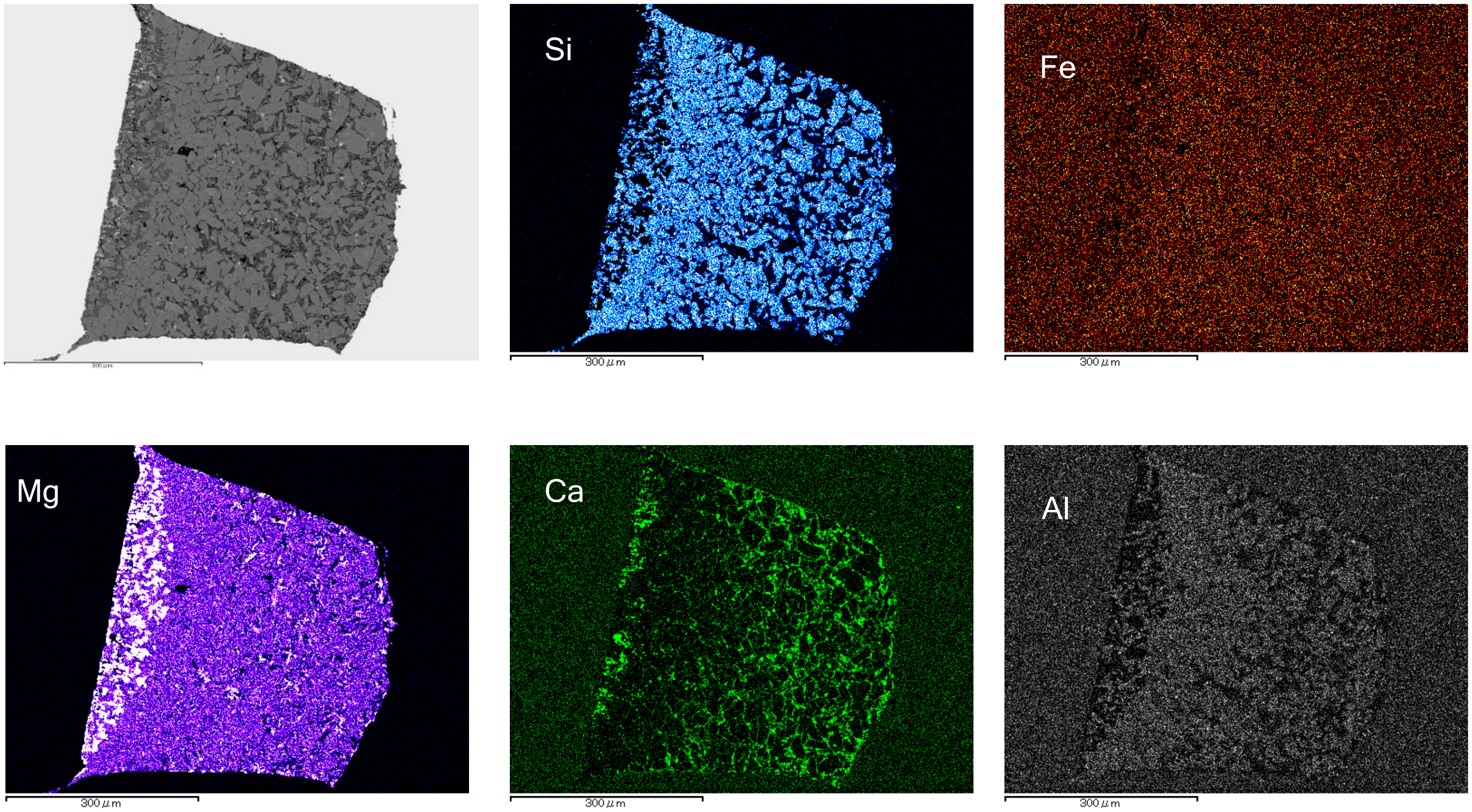

Supplementary Figure S3.7. Elemental mapping images and electron microscope image (upper left) of recovered sample at 26 GPa and 1590 °C (duration time: 15 minutes).

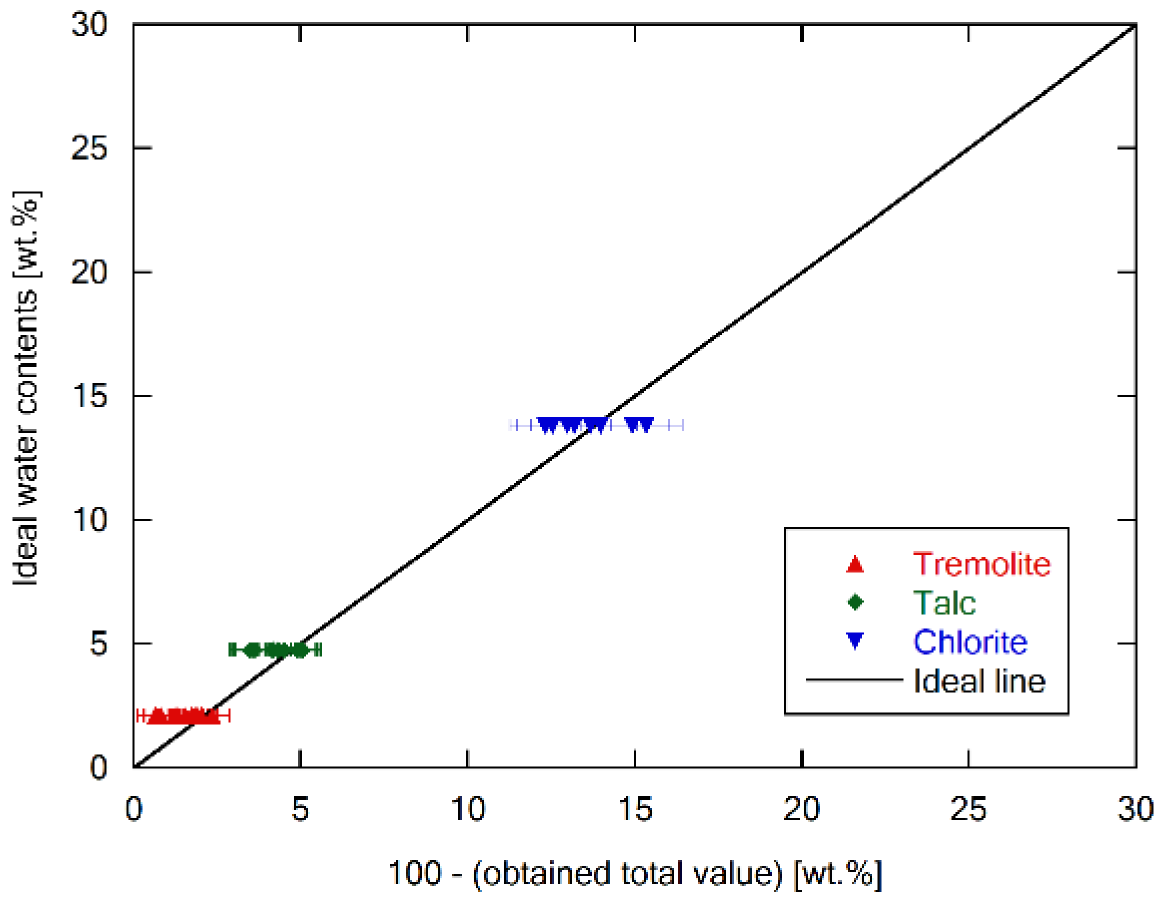

Supplementary Figure S4. Relationship between the water content of hydrous minerals and the difference between ideal total value (100 wt.%) and obtained total mass by EDS analysis.

Red triangles, green diamonds, and blue inverted triangles are tremolite ($Ca_2(Mg,Fe)_5Si_8O_{22}(OH)_2$,; H_2O = 2.12 wt.%), talc ($Mg_3Si_4O_{10}(OH)_2$; H_2O = 4.75 wt.%) and chlorite $(Mg,Fe^{2+})_{10}Al_2[Al_2Si_6O_{20}](OH)_{16}$; H_2O = 13.82 wt.%,) respectively. Solid line indicates the one-to-one relationship and each data is on the line.

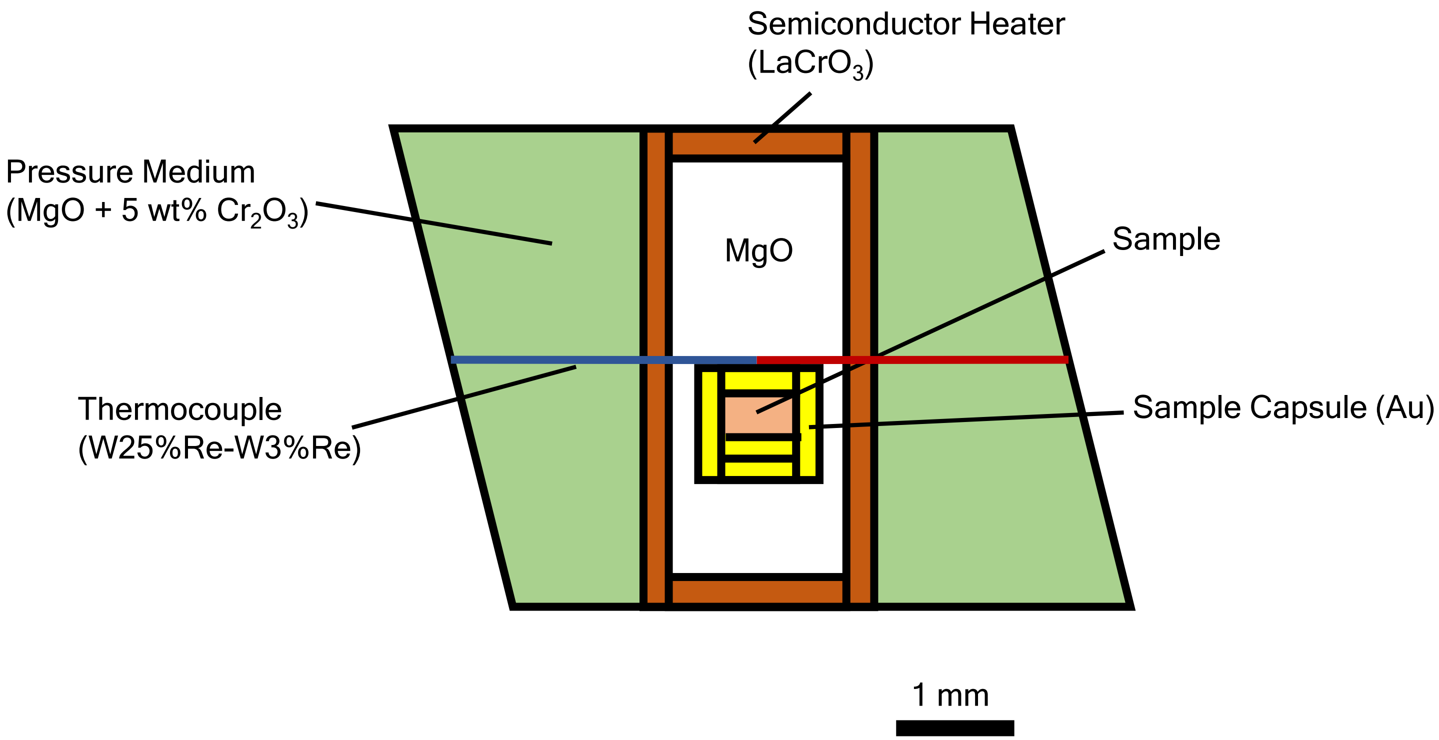

Supplementary Figure S5. Cross-section of the high-pressure cell assembly.

Supplementary Table S1.1 Chemical composition of recovered sample at 23.5 GPa and 1400°C (duration time: 50 minutes).

	Bridgmanite (wt.%)	Ferro-periclase (wt.%)	$CaSiO_3$-perovskite (wt.%)	Melt (wt.%)		Bridgmanite (No. of ions)	Ferro-periclase (No. of ions)	$CaSiO_3$-perovskite (No. of ions)	Melt (No. of ions)
SiO_2	52.6 (0.5)	-	49.2 (2.3)	17.3 (1.0)	Si	7.14	-	7.78	3.83
Al_2O_3	5.2 (0.1)	-	1.2 (0.2)	1.5 (0.1)	Al	0.82	-	0.23	0.39
FeO*	9.0 (0.3)	3.0 (0.2)	0.5 (1.6)	7.9 (0.3)	Fe	0.92	0.41	0.01	1.32
MgO	34.9 (0.4)	97.4 (0.7)	2.5 (4.7)	36.1 (2.0)	Mg	7.06	23.59	0.60	11.92
CaO	-	-	43.7 (6.4)	7.7 (1.3)	Ca	-	-	7.39	1.83
total	101.7	100.4	97.2	70.6	total cation	15.94	24.00	16.01	19.29
N	10	10	5	10					

*The valence of FeO is unknown. N is a number of measured crystals. Inside of parentheses is standard deviation. Total anion of each mineral is 24.

Supplementary Table S1.2 Chemical composition of recovered sample at 23.5 GPa and 1500°C (duration time: 60 minutes).

	Bridgmanite (wt.%)	Ferro-periclase (wt.%)	$CaSiO_3$-perovskite (wt.%)	Melt (wt.%)		Bridgmanite (No. of ions)	Ferro-periclase (No. of ions)	$CaSiO_3$-perovskite (No. of ions)	Melt (No. of ions)
SiO_2	52.6 (0.4)	-	38.9 (6.6)	15.8 (2.0)	Si	7.16	-	7.13	3.57
Al_2O_3	5.0 (0.2)	-	1.3 (0.2)	1.3 (0.2)	Al	0.80	-	0.29	0.34
FeO*	8.5 (0.3)	3.0 (0.2)	0.5 (0.8)	7.7 (0.6)	Fe	0.96	0.40	0.11	1.31
MgO	34.7 (0.2)	97.9 (1.2)	6.8 (4.1)	33.9 (3.4)	Mg	7.03	23.60	1.76	11.41
CaO	-	-	37.3 (3.5)	12.1 (3.8)	Ca	0.00	-	7.43	2.99
total	100.7	100.9	84.8	70.8	total cation	15.95	24.00	16.72	19.6
N	10	7	3	10					

*The valence of FeO is unknown. N is a number of measured crystals. Inside of parentheses is standard deviation. Total anion of each mineral is 24.

Supplementary Table S1.3 Chemical composition of recovered sample at 23.5 GPa and 1600°C (duration time: 15 minutes).

	Bridgmanite (wt.%)	Ferro-periclase (wt.%)	$CaSiO_3$-perovskite (wt.%)	Melt (wt.%)		Bridgmanite (No. of ions)	Ferro-periclase (No. of ions)	$CaSiO_3$-perovskite (No. of ions)	Melt (No. of ions)
SiO_2	54.0 (0.7)	-	51.7	22.9 (2.3)	Si	7.18	-	7.96	4.47
Al_2O_3	5.2 (0.2)	-	0.8	2.2 (0.4)	Al	0.82	-	0.16	0.51
FeO*	8.6 (0.5)	5.0 (0.3)	-	7.9 (0.6)	Fe	0.22	0.67	-	1.16
MgO	35.9 (0.5)	98.9 (1.2)	1.2	37.0 (3.6)	Mg	7.11	23.33	0.28	10.80
CaO	0.2 (0.2)	-	45.8	8.4 (3.4)	Ca	0.02	-	7.56	1.75
total	103.8	103.9	99.5	78.3	total cation	15.34	24.00	15.96	18.69
N	10	5	1	10					

*The valence of FeO is unknown. N is a number of measured crystals. Inside of parentheses is standard deviation. Total anion of each mineral is 24.

Supplementary Table S1.4 Chemical composition of recovered sample at 26 GPa and 1300°C (duration time: 100 minutes).

	Bridgmanite (wt.%)	Ferro-periclase (wt.%)	$CaSiO_3$-perovskite (wt.%)	Melt (wt.%)		Bridgmanite (No. of ions)	Ferro-periclase (No. of ions)	$CaSiO_3$-perovskite (No. of ions)	Melt (No. of ions)
SiO_2	52.7 (0.5)	0.3 (1.3)	50.0 (0.6)	11.1 (2.3)	Si	7.10	0.04	7.87	2.79
Al_2O_3	5.1 (0.3)	-	0.9 (0.1)	1.2 (0.3)	Al	0.82	-	0.17	0.36
FeO*	10.0 (0.7)	2.7 (0.3)	-	8.7 (1.0)	Fe	1.01	0.35	-	1.68
MgO	35.1 (0.4)	100.1 (1.3)	0.9 (0.4)	34.1 (1.9)	Mg	7.05	23.57	0.21	12.92
CaO	-	-	46.2 (0.8)	9.0 (2.3)	Ca	-	-	7.78	2.46
total	102.8	103.0	98.0	64.2	total cation	15.98	23.96	16.03	20.21
N	10	10	10	10					

*The valence of FeO is unknown. N is a number of measured crystals. Inside of parentheses is standard deviation. Total anion of each mineral is 24.

Supplementary Table S1.5 Chemical composition of recovered sample at 26 GPa and 1400°C (duration time: 60 minutes).

	Bridgmanite (wt.%)	Ferro-periclase (wt.%)	$CaSiO_3$-perovskite (wt.%)	Melt (wt.%)		Bridgmanite (No. of ions)	Ferro-periclase (No. of ions)	$CaSiO_3$-perovskite (No. of ions)	Melt (No. of ions)
SiO_2	53.1 (0.5)	0.1 (0.2)	50.0 (0.3)	14.9 (1.7)	Si	7.14	0.01	7.88	3.41
Al_2O_3	5.2 (0.1)	-	1.2 (0.1)	1.6 (0.2)	Al	0.82	-	0.23	0.42
FeO*	9.2 (0.3)	3.6 (0.2)	-	9.1 (0.6)	Fe	0.93	0.49	-	1.57
MgO	35.4 (0.4)	99.1 (0.7)	1.0 (0.4)	35.6 (3.9)	Mg	7.09	23.50	0.25	12.12
CaO	-	-	45.3 (1.0)	8.4 (2.2)	Ca	-	-	7.65	2.06
total	102.9	102.7	97.6	69.5	total cation	15.97	23.99	16.01	19.58
N	10	10	3	10					

*The valence of FeO is unknown. N is a number of measured crystals. Inside of parentheses is standard deviation. Total anion of each mineral is 24.

Supplementary Table S1.6 Chemical composition of recovered sample at 26 GPa and 1400°C (duration time: 120 minutes).

	Bridgmanite (wt.%)	Ferro-periclase (wt.%)	$CaSiO_3$-perovskite (wt.%)	Melt (wt.%)		Bridgmanite (No. of ions)	Ferro-periclase (No. of ions)	$CaSiO_3$-perovskite (No. of ions)	Melt (No. of ions)
SiO_2	53.6 (0.5)	-	50.3 (1.4)	11.6 (3.5)	Si	7.15	-	7.90	2.88
Al_2O_3	5.1 (0.3)	-	1.1 (0.2)	1.5 (0.4)	Al	0.8	-	0.20	0.44
FeO*	9.1 (0.7)	3.8 (1.1)	-	7.6 (0.6)	Fe	0.91	0.51	-	1.44
MgO	35.8 (0.6)	98.9 (0.9)	0.9 (2.0)	33.8 (1.8)	Mg	7.12	23.43	0.22	12.75
CaO	-	-	45.6 (2.0)	9.6 (2.7)	Ca	-	-	7.67	2.65
total	103.6	102.8	97.9	64.0	total cation	15.98	23.94	16.00	20.16
N	10	10	10	10					

*The valence of FeO is unknown. N is a number of measured crystals. Inside of parentheses is standard deviation. Total anion of each mineral is 24.

Supplementary Table S1.7 Chemical composition of recovered sample at 26 GPa and 1500°C (duration time: 30 minutes).

	Bridgmanite (wt.%)	Ferro-periclase (wt.%)	$CaSiO_3$-perovskite (wt. %)	Melt (wt.%)		Bridgmanite (No. of ions)	Ferro-periclase (No. of ions)	$CaSiO_3$-perovskite (No. of ions)	Melt (No. of ions)
SiO_2	54.3 (0.6)	-	50.5 (0.6)	14.9 (3.8)	Si	7.16	-	7.84	3.39
Al_2O_3	5.2 (0.2)	-	1.3 (0.0)	1.6 (0.5)	Al	0.80	-	0.24	0.42
FeO*	9.1 (0.4)	3.8 (0.1)	0.4 (0.4)	7.9 (0.7)	Fe	0.90	0.50	0.04	1.36
MgO	36.2 (0.4)	100.1 (1.5)	2.1 (1.8)	34.5 (3.5)	Mg	7.11	23.50	0.48	11.81
CaO	-	-	44.7 (2.0)	11.0 (3.2)	Ca	-	-	7.44	2.74
total	104.7	104.0	99.0	70.0	total cation	15.98	24.00	16.04	19.72
N	10	9	2	10					

*The valence of FeO is unknown. N is a number of measured crystals. Inside of parentheses is standard deviation. Total anion of each mineral is 24.

Supplementary Table S1.8 Chemical composition of recovered sample at 26 GPa and 1590°C (duration time: 15 minutes).

	Bridgmanite (wt.%)	Ferro-periclase (wt.%)	$CaSiO_3$-perovskite (wt.%)	Melt (wt.%)		Bridgmanite (No. of ions)	Ferro-periclase (No. of ions)	$CaSiO_3$-perovskite (No. of ions)	Melt (No. of ions)
SiO_2	53.3 (0.5)	-	50.0 (0.5)	14.3 (3.8)	Si	7.15	-	7.86	3.25
Al_2O_3	5.4 (0.2)	-	1.6 (0.0)	1.7 (0.4)	Al	0.86	-	28	0.46
FeO*	8.9 (0.4)	4.1 (0.3)	-	8.3 (0.5)	Fe	0.90	0.55	-	1.43
MgO	35.4 (0.4)	99.1 (0.9)	-	36.6 (5.6)	Mg	7.07	23.45	-	12.38
CaO	-	-	47.0 (0.6)	9.3 (2.4)	Ca	-	-	7.84	2.29
total	103.0	103.2	99.0	70.3	total cation	15.98	24.00	15.98	19.80
N	10	10	2	10					

*The valence of FeO is unknown. N is a number of measured crystals. Inside of parentheses is standard deviation. Total anion of each mineral is 24.

Supplementary References

1. Ito, E. & Takahashi, E. Melting of peridotite at uppermost lower-mantle conditions. *Nature* **328**, 514–517 (1987).

2. Iwamori, H. Phase relations of peridotites under H_2O-saturated conditions and ability of subducting plates for transportation of H_2O. *Earth Planet. Sci. Lett.* **227**, 57-71 (2004).

3. Kawamoto, T. Hydrous phase stability and partial melt chemistry in H2O-saturated KLB-1 peridotite up to the uppermost lower mantle conditions. Phys. Earth Planet. Inter. 143–144, 387–395 (2004).

4. Stacey, F. D. A thermal model of the Earth. *Phys. Earth Planet. Inter.* **15**, 341–348 (1977).

5. Brown, J. M. & Shankland, T. J. Thermodynamic parameters in the Earth as determined from seismic profiles. *Geophys. J. Int.* **66**, 579–596 (1981).
